# Genetic and Functional Sequence Variants of the SIRT3 Gene Promoter in Myocardial Infarction

**DOI:** 10.1371/journal.pone.0153815

**Published:** 2016-04-14

**Authors:** Xiaoyun Yin, Shuchao Pang, Jian Huang, Yinghua Cui, Bo Yan

**Affiliations:** 1 Department of Medicine, Shandong University School of Medicine, Jinan, Shandong, China; 2 Shandong Provincial Key Laboratory of Cardiac Disease Diagnosis and Treatment, Affiliated Hospital of Jining Medical University, Jining Medical University, Jining, Shandong, China; 3 Division of Cardiology, Affiliated Hospital of Jining Medical University, Jining Medical University, Jining, Shandong, China; 4 Shandong Provincial Sino-US Cooperation Research Center for Translational Medicine, Affiliated Hospital of Jining Medical University, Jining Medical University, Jining, Shandong, China; Boston University School of Medicine, UNITED STATES

## Abstract

Coronary artery disease (CAD), including myocardial infarction (MI), is a common complex disease that is caused by atherosclerosis. Although a large number of genetic variants have been associated with CAD, only 10% of CAD cases could be explained. It has been proposed that low frequent and rare genetic variants may be main causes for CAD. SIRT3, a mitochondrial deacetylase, plays important roles in mitochondrial function and metabolism. Lack of SIRT3 in experimental animal leads to several age-related diseases, including cardiovascular diseases. Therefore, SIRT3 gene variants may contribute to the MI development. In this study, SIRT3 gene promoter was genetically and functionally analyzed in large cohorts of MI patients (n = 319) and ethnic-matched controls (n = 322). Total twenty-three DNA sequence variants (DSVs) were identified, including 10 single-nucleotide polymorphisms (SNPs). Six novel heterozygous DSVs, g.237307A>G, g.237270G>A, g.237023_25del, g.236653C>A, g.236628G>C, g.236557T>C, and two SNPs g.237030C>T (rs12293349) and g.237022C>G (rs369344513), were identified in nine MI patients, but in none of controls. Three SNPs, g.236473C>T (rs11246029), g.236380_81ins (rs71019893) and g.236370C>G (rs185277566), were more significantly frequent in MI patients than controls (P<0.05). These DSVs and SNPs, except g.236557T>C, significantly decreased the transcriptional activity of the SIRT3 gene promoter in cultured HEK-293 cells and H9c2 cells. Therefore, these DSVs identified in MI patients may change SIRT3 level by affecting the transcriptional activity of SIRT3 gene promoter, contributing to the MI development as a risk factor.

## Introduction

Coronary artery disease (CAD) is a common complex disease, which is a leading cause of human death around the world. Myocardial infarction (MI) is a specific type of CAD. Atherosclerosis, an inflammatory and metabolic disease, is the main cause for CAD and MI. Known risk factors include hypertension, smoking, diabetes, hyperlipoproteinemia and hypercholesterolemia. Though genome-wide association studies have identified more than 50 genetic loci to CAD, these genetic variants account for only 10% of cases [1–3]. To date, genetic causes and underlying molecular mechanisms for CAD remain largely unclear. It has been hypothesized that low frequency and rare variants with large effects may account for some of the missing heritability for CAD. Recently, epigenetic factors have been suggested to contribute to aging and age-associated diseases [4].

Sirtuins (SIRTs), a family of nicotinamide adenine dinucleotide (NAD+)-dependent deacetylases, have been involved in a wide range of cellular processes, including aging, calorie restriction, stress resistance, apoptosis, inflammation, mitochondrial function and circadian clock. Dysfunctional SIRTs have been implicated in a variety of age-related diseases, including cardiovascular diseases [5,6]. Seven SIRTs, SIRT1-7, have been identified in mammals. SIRT3 is localized in mitochondria and has been extensively studied. Increased expression of SIRT3 gene has been associated with extended lifespan of humans [7–9]. SIRT3 regulates the enzymes involving in the respiratory chain, ATP production, fatty acid oxidation, tricarboxylic acid cycle and urea cycle, and reduces levels of reactive oxygen species (ROS) and oxidative stress [10–12]. Tissue-specific SIRT3 deletion studies suggest its non-tissue-autonomous roles [13]. Therefore, SIRT3 plays an important role in regulating mitochondrial function and metabolism. Lack of SIRT3 in experimental animal leads to several age-related diseases, such as cancer, metabolic syndrome, cardiovascular disease, and neurodegenerative diseases [14].

SIRT3 has been demonstrated to protect cardiomyocytes from aging, oxidative stress and dysregulated metabolism, and suppresses cardiac hypertrophy [15,16]. In cultured cardiomyocytes, SIRT3 inhibits myocardial reperfusion injury and reduces oxidative stress-induced apoptosis [17,18]. SIRT3 knockdown increases the susceptibility of cultured cardiomyocytes and adult hearts to ischemia-reperfusion injury [19]. In SIRT3-knockout mice, ROS levels are increased in the cardiomyocytes, indicating that SIRT3 prevents cellular ROS accumulation in the heart [20]. Mitochondrial permeability is also increased in the heart, leading to mitochondrial dysfunction [21]. SIRT3 is necessary for bone marrow cell-mediated cardiac repair in post-myocardial infarction [22]. In human endothelial cells, SIRT3 mediates the cellular response to hypoxia and protects the cells from high glucose-induced cytotoxicity [23,24]. In humans and animals, SIRT3 has been shown to regulate lipid metabolism and reduce lipid accumulation in diverse tissues, including heart [25–27]. SIRT3 has recently been reported to be involved in inflammation as well as platelet aging and thrombosis [28,29]. In addition, overexpressed SIRT3 enhances autophagy, which plays an important role in the development of cardiovascular diseases, including atherosclerosis, cardiac ischemia/reperfusion, cardiomyopathy and heart failure [30–32]. Therefore, SIRT3 may be involved in development of cardiovascular diseases through pathways of metabolism, inflammation and others.

SIRT3 gene expression has been studied and reported in several human diseases. SIRT3 gene expression is increased in hepatocellular carcinoma cells and human embryonic kidney cells (HEK-293) under hyperglycemic conditions [33]. Decreased SIRT3 expression has been reported in the patients with metabolic syndrome, non-alcoholic fatty liver disease, pulmonary arterial hypertension and type 2 diabetes [34–37]. Downregulated and upregulated SIRT3 gene expression has also been observed in different cancer tissues, depending on the cell and cancer types [38,39]. However, molecular mechanisms by which SIRT3 gene expression is changed have not been reported. We speculated that the DNA sequence variants (DSVs) within the regulatory regions of the SIRT3 gene may account for the changed SIRT3 gene expression. In this study, we genetically and functionally analyzed the promoter region of the SIRT3 gene in large cohorts of MI patients and healthy controls.

## Materials and Methods

### Patients and controls

All MI patients (n = 319, male 236, female 89, age range from 33 to 90 years, median age 60.03 years) were recruited between July, 2012 to November, 2014, from Cardiac Care Unit, Division of Cardiology, Affiliated Hospital of Jining Medical University, Jining Medical University, Jining, Shandong, China. All MI patients were diagnosed according to international guideline criteria: ischemic symptoms, ECG changes (ST-segment elevation or depression), typical rise of biochemical markers of myocardial necrosis (troponin or creatine kinase-MB), or coronary angioplasty. Patients with valvular heart disease, cardiomyopathy, myocarditis, or postrevascularization of the coronary arteries were excluded from this study. Ethnic-matched healthy controls (n = 322, male 172, female 150, age range from 21 to 82 years, median age 48.97 years) were recruited from the same hospital. Controls with familial MI history were excluded. In this study, 342 MI patients and 346 controls were initially recruited. Since DNA sequencing for some samples were failed, total 319 MI patients and 322 controls were finally included. The research was carried out according to the principles of the Declaration of Helsinki. This study was approved by the Human Ethic Committee of Affiliated Hospital of Jining Medical University. Written informed consents were obtained.

### DNA Sequence analysis

Peripheral leukocytes were isolated and genomic DNAs were extracted. SIRT3 gene promoter (1200bp upstream to the transcription start site) was sequenced and analyzed. Two overlapped DNA fragments, 764bp (-1200bp~-437bp) and 678bp (-498bp~+180bp), were generated by PCR. PCR primers were designed based on genomic sequence of the human SIRT3 gene (NCBI, NC_000011.10), which were shown in Table 1. PCR products were bi-directionally sequenced with Applied Biosystems 3500xL genetic analyzer. The DNA sequences were then aligned with the wild type sequence of the SIRT3 gene promoter.

**Table 1:** PCR primers for the SIRT3 gene promoter.

PCR primers	Sequences	Location	Position	Products
Sequencing				
SIRT3-F1	5′-AAGCACGAATCACCAACATG-3′	237562	-1200	764bp
SIRT3-R1	5′-CCCACACTCTTTGACGCCT-3'	236799	-437	
SIRT3-F2	5′-CGGCGTTTGCAGCCCACTTC-3'	236860	-498	678bp
SIRT3-R2	5′-CCCGCACTCACATCGTCCCT-3'	236183	+180	
Functioning				
SIRT3-F	5′-(KpnI)-CTTGGCCCTCGTGGTCTGTC-3'	237361	-999	1179bp
SIRT3-R	5′-(HindIII)-GCGCAGTCCAAGGAGTCCTC-3'	236183	+180	

PCR primers are designed based on the genomic DNA sequence of the SIRT3 gene (NC_000011.10). The transcription start site (TSS) is at the position of 236362 (+1).

### Functional analysis of the DSVs by dual-luciferase reporter assays

Expression vectors were constructed by subcloning wild type and variant SIRT3 gene promoters into luciferase reporter vector (pGL3-basic) and dual-luciferase activities were examined. Briefly, DNA fragments of wild type and variant SIRT3 gene promoters (1179bp, from -999bp to +180bp to the transcription start site) were generated by PCR and then subcloned into KpnI and Hind III sites of pGL3-basic to generate expression vectors. The PCR primers with KpnI or HindIII sites were shown in Table 1. Designated expression vectors were transiently transfected into human embryonic kidney cells (HEK-293, from ATCC, CRL-1573) or rat cardiomyocyte line cells (H9c2, from ATCC, CRL-1446). After 48 hours, the luciferases activities of the transfected cells were measured using dual-luciferase reporter assay system on a Promega Glomax 20/20 luminometer. Expression vector pRL-TK expressing renilla luciferase was used as an internal control for transfection. Empty vector pGL3-basic was used as a negative control. The transcriptional activities of the SIRT3 gene promoter were represented as ratios of luciferase activities over renilla luciferase activities. All the experiments were repeated three times independently, in triplicate.

### Statistical analysis

The quantitative data were represented as mean ± SEM and compared by a standard Student's t-test. DSV frequencies in MI patients and controls were analyzed and compared with SPSS v13.0. P<0.05 was considered statistically significant.

## Results

### Identified DSVs of the SIRT3 gene promoter

**Fig 1:**
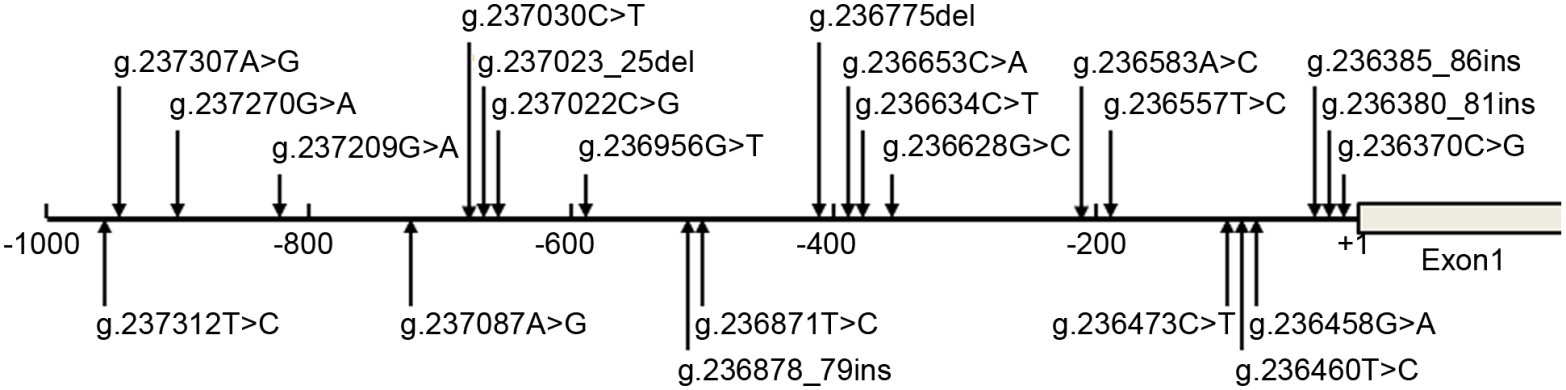
Locations of the DSVs within the SIRT3 gene promoter in MI patients and controls. The numbers represents the genomic DNA sequences of the human SIRT3 gene (Genebank accession number NC_000011.10). The transcription start site is at the position of 236362 in the first exon.

**Fig 2:**
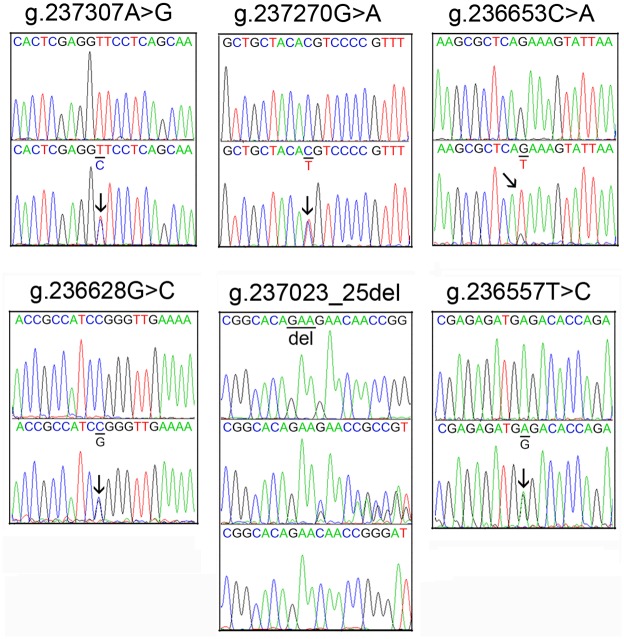
Chromatograms of the novel DSVs within the SIRT3 gene promoter identified in MI patients. All the DSVs are shown in forward orientations. For DSVs g.237307A>G, g.237270G>A, g.236653C>A, g.236628G>C and g.236557T>C, top panels show wild type and bottom panels heterozygous DSVs, which are marked with arrows. For the deletion DSV g.237023_25del, top panel shows wild type, middle panel heterozygous and bottom panel cloning DNA sequences. The deletion is underlined and labeled.

In this population, total 23 DSVs, including 10 SNPs, were identified. The locations and frequencies of the DSVs and SNPs were shown in Fig 1 and Table 2. Five novel heterozygous DSVs (g.237307A>G, g.237270G>A, g.236653C>A, g.236628G>C, g.236557T>C), one novel heterozygous deletion DSV (g.237023_25del) and two SNPs [g.237030C>T (rs12293349) and g.237022C>G (rs369344513)] were identified in 9 MI patients, but in none of controls. Chromatograms of these novel DSVs were shown in Fig 2. Three SNPs, g.236473C>T (rs11246029), g.236380_81ins (rs71019893) and g.236370C>G (rs185277566) were more significantly frequent in MI patients than controls (P<0.05). More interestingly, SNPs g.236380_81ins (rs71019893) and g.236370C>G (rs185277566) were closely linked together with the same frequencies.

**Table 2:** The DSVs within the SIRT3 gene promoter in MI patients and controls.

DSVs	Genotypes	Location^1^	Controls (n = 322)	AMI (n = 319)	P value
g.237312T>C (rs3817629)	TT	-950bp	5	8	0.591
	TC		87	92	
	CC		230	219	
g.237307A>G	AG	-945bp	0	1	-
g.237270G>A	GA	-908bp	0	1	-
g.237209G>A (rs56312618)	GA	-847bp	2	5	0.254
g.237087A>G (rs1045288)	AA	-725bp	5	8	0.591
	AG		87	92	
	GG		230	219	
g.237030C>T (rs12293349)	CT	-668bp	0	1	-
g.237023_25delTTC	TTC/-	-661bp	0	1	-
g.237022C>G (rs369344513)	CG	-660bp	0	2	-
g.236956G>T	GT	-594bp	1	0	-
g.236878_79insG	-/G	-516bp	1	0	-
g.236871T>C (rs2272563)	TT	-509bp	5	8	0.591
	TC		87	92	
	CC		230	219	
g.236775delC (rs369178836)	C/-	-414bp	13	9	0.410
g.236653C>A	CA	-291bp	0	1	-
g.236634C>T	CT	-272bp	1	0	-
g.236628G>C	GC	-266bp	0	1	-
g.236583A>C	AC	-217bp	1	0	-
g.236557T>C	TC	-195bp	0	1	-
g.236473C>T (rs11246029)	CC	-111bp	13	15	0.035
	CT		54	79	
	TT		255	225	
g.236460T>C	TC	-98bp	1	0	-
g.236458G>A	GA	-96bp	1	0	-
g.236385_86ins29bp	-/-	-23bp	318	317	0.373
(CGGCGCCCCGCCCGCGGCGCCCCGGCCCC)	-/29bp		4	1	
	29bp/29bp		0	1	
g.236380_81ins14bp (rs71019893)	-/-	-19bp	12	19	0.008
(CCCGCGGCGCCCCG)	-/14bp		56	83	
	14bp/14bp		254	217	
g.236370C>G (rs185277566)	CC	-8bp	12	19	0.008
	CG		56	83	
	GG		254	217	

^1^, DSVs are located upstream (-) to the transcription start site of SIRT3 gene at 236362 of NC_000011.10.

**Fig 3:**
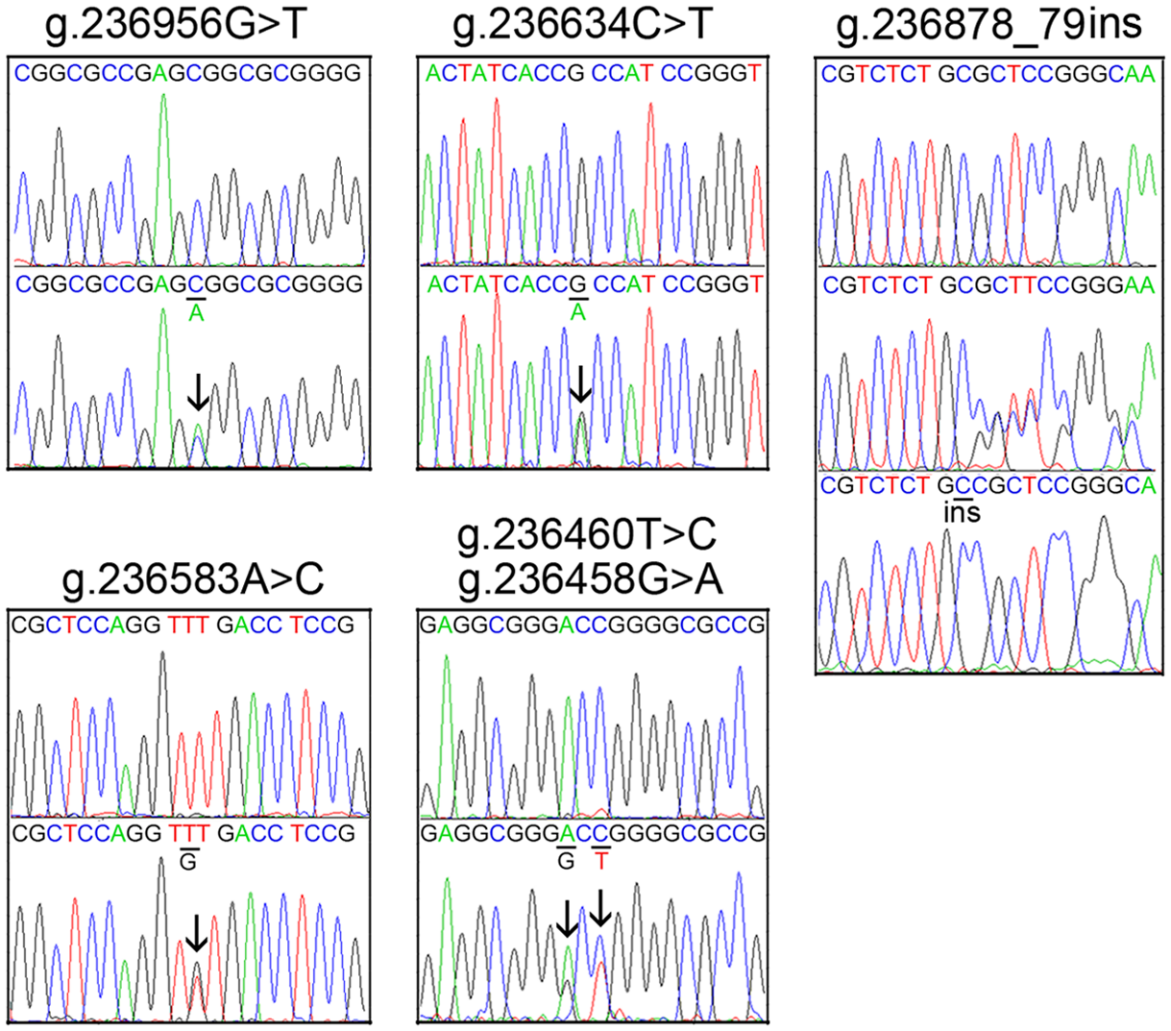
Chromatograms of the DSVs within the SIRT3 gene promoter only identified in controls. All the novel DSVs are depicted in forward orientations. The DSVs g.236956G>T, g.236634C>T, g.236583A>C, g.236460T>C and g.236458G>A, top panels show wild type and bottom panels heterozygous DSVs, which are marked with arrows. For the insertion DSV g.236878_79ins, top panel shows wild type, middle panel heterozygous and bottom panel cloning DNA sequences. The insertion is underlined and labeled.

Six novel heterozygous DSVs, g.236956G>T, g.236878_79ins, g.236634C>T, g.236583A>C, g.236460T>C and g.236458G>A, were only found in two controls. Chromatograms of these novel DSVs were shown in Fig 3. The rest five SNPs [g.237312T>C (rs3817629), g.237209G>A (rs56312618), g.237087A>G (rs1045288), g.236871T>C (rs2272563) and g.236775delC (rs369178836)] and one deletion DSV (g.236385_86ins), were found in both VSD patients and controls with similar frequencies (P>0.05).

### Putative binding sites for transcription factors affected by DSVs

The SIRT3 gene promoter was analyzed with JASPAR program (http://jaspar.genereg.net/). The DSVs identified in MI patients may abolish, create and modify the putative binding sites for transcription factors. For example, the DSV g.237307A>G may abolish the binding sites for SPIB [Spi-B transcription factor (Spi-1/PU.1 related)] and myeloid zinc finger 1, and create the binding sites for estrogen-related receptor beta and nuclear receptor subfamily 4 group A member 2. The DSV g.237270G>A may abolish the binding sites for hairy/enhancer-of-split related with YRPW motif protein 2 and the dimer between hypoxia-inducible factor 1-alpha and aryl hydrocarbon receptor nuclear translocator, and create the binding sites for doublesex and Mab-3 related transcription factor 3, HLTF (helicase-like transcription factor) and MEIS1. The SNP g.237030C>T (rs12293349) may create the binding sites for zinc finger and BTB domain containing 7B, insulinoma-associated 1, MEIS1 and SOX10 [SRY (sex determining region Y) box 10]. The DSV g.237023_25del may abolish the binding site for SPIB. The SNP g.237022C>G (rs369344513) may abolish the binding site for SPIB and create the binding site for FOXD2 (forkhead box factor D2). The DSV g.236653C>A may abolish the binding site for SOX10 and create the binding sites for caudal-related homeobox transcription factor 1, homeobox factors, motor neuron and pancreas homeobox 1, NKX6-1 (NK6 transcription factor related, locus 1), NKX6-2 and NK-related factor NOTO. The DSV g.236628G>C may abolish the binding sites for E74-Like factor 5, Ets-variant gene 5, chorion-specific transcription factor GCMb, neurogenic differentiation factor 2, SAM pointed domain containing ETS transcription factor and THAP domain-containing protein 1. The DSV g.236557T>C may abolish the binding sites for FOS transcription factor L1, transcription factor JUND and MEIS1 and create a binding site for HLTF. The SNP g.236473C>T (rs11246029) may abolish the binding sites for zinc finger protein 263, SP4 (specificity protein 4), KLF14 (Kruppel-like factor 14) and SP8, and create a binding site for E2F transcription factor 6. The SNP g.236380_81ins (rs71019893) may abolish the binding sites for SP1, SP2, KLF5 and KLF16, and create binding sites for TFAP2A (transcription factor AP-2 alpha), TFAP2B, TFAP2C, ZIC1 (zinc finger transcription factor 1) and ZIC4. The SNP g.236370C>G (rs185277566) may abolish the binding sites for early growth response protein 1, KLF16, SP1, SP2 and SP3.

### DSVs Effects on transcriptional activity of SIRT3 gene promoter

**Fig 4:**
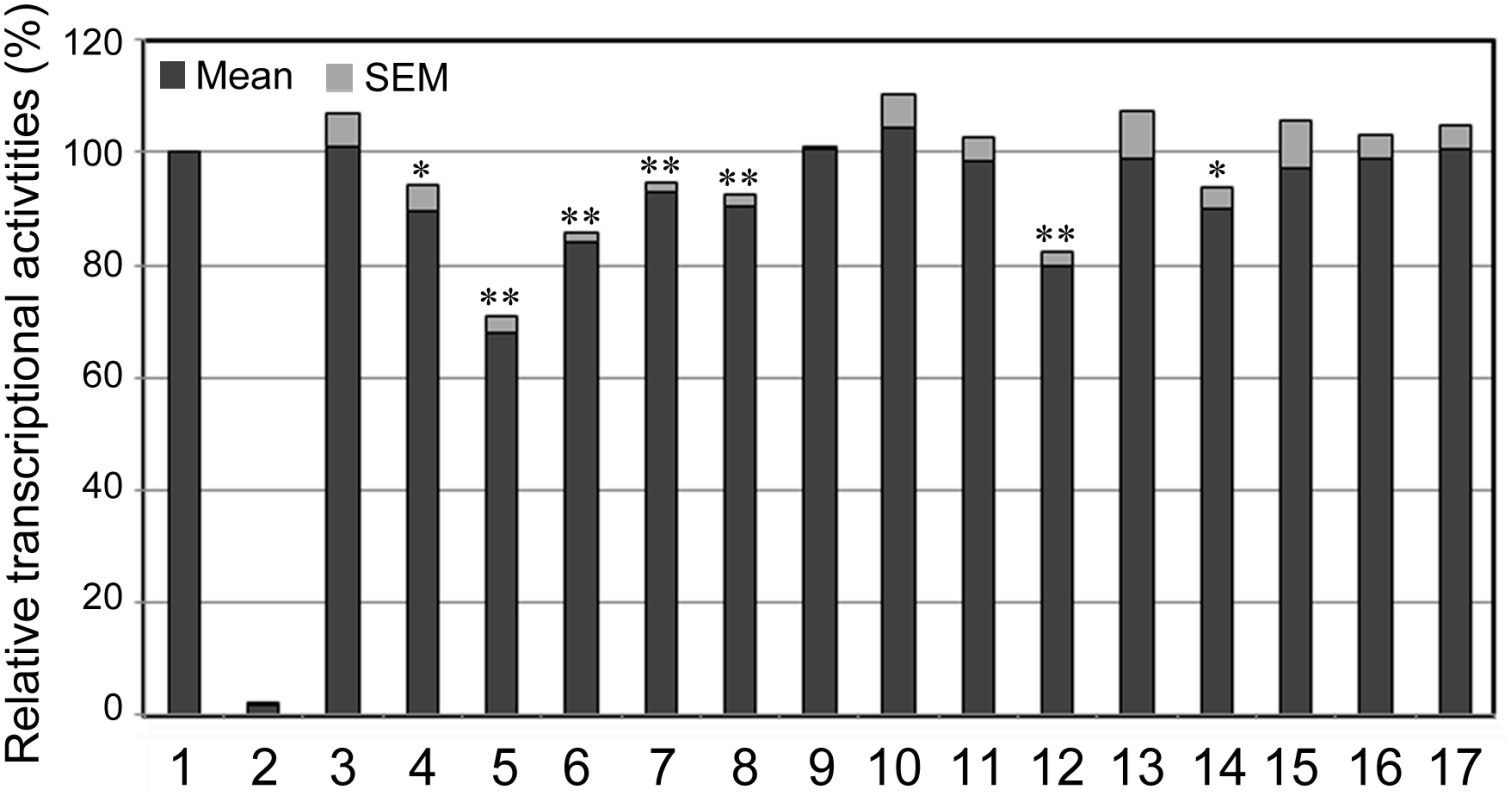
Relative transcriptional activities of wild type and variant SIRT3 gene promoters in H9c2 cells. Wild type and variant SIRT3 gene promoters were cloned into reporter gene vector pGL3 and transfected into H9c2 cells. Dual-luciferase activities were assayed and relative activities of SIRT3 gene promoters were obtained. Transcriptional activity of the wild type SIRT3 gene promoter was designed as 100%. Lanes 1, pGL3-WT; 2, pGL3 control; 3, pGL3-g.237312T>C (rs3817629); 4, pGL3-g.237307A>G; 5, pGL3-g.237270G>A + g.236370C>G + g.236380_81ins; 6, pGL3-g.237030C>T (rs12293349); 7, pGL3-g.237023_25del; 8, pGL3-g.237022C>G (rs369344513); 9, pGL3-g.236956G>T; 10, pGL3-g.236878_79Ins; 11, pGL3-g.236775del (rs369178836); 12, pGL3-g.236653C>A + g.236473C>T; 13, pGL3-g.236634C>T; 14, pGL3-g.236628G>C; 15, pGL3-g.236583A>C; 16, pGL3-g.236557T>C; 17, pGL3-g.236460T>C. *, P<0.05; **, P<0.01.

Wild type and variant SIRT3 gene promoters were cloned into luciferase reporter vector (pGL3-basic) to generate expression constructs. After transfected into cultured cells, rat cardiomyocyte cells (H9c2) and human embryonic-kidney cells (HEK-293), dual-luciferase activities were assayed to examine the transcriptional activities of the SIRT3 gene promoter. In H9c2 cells, the DSVs only identified in MI patients or the DSVs found in MI patients with higher frequencies, g.237307A>G, g.237270G>A+g.236370C>G+g.236380_81ins, g.237030C>T (rs12293349), g.237023_25del, g.237022C>G (rs369344513), g.236653C>A+g.236473C>T and g.236628G>C, significantly repressed the transcriptional activities of the SIRT3 gene promoter to different extents (P<0.05, Fig 4). However, the DSV g.236557T>C, which was identified in a MI patient, did not significantly alter the transcriptional activity of the SIRT3 gene promoter (P>0.05). As expected, the DSVs only identified in controls or the DSVs found in MI patients and controls with similar frequencies, g.237312T>C (rs3817629), g.236956G>T, g.236878_79ins, g.236775del (rs369178836), g.236634C>T, g.236583A>C and g.236460T>C, did not significantly changed the transcriptional activity of the SIRT3 gene promoter (P>0.05).

**Fig 5:**
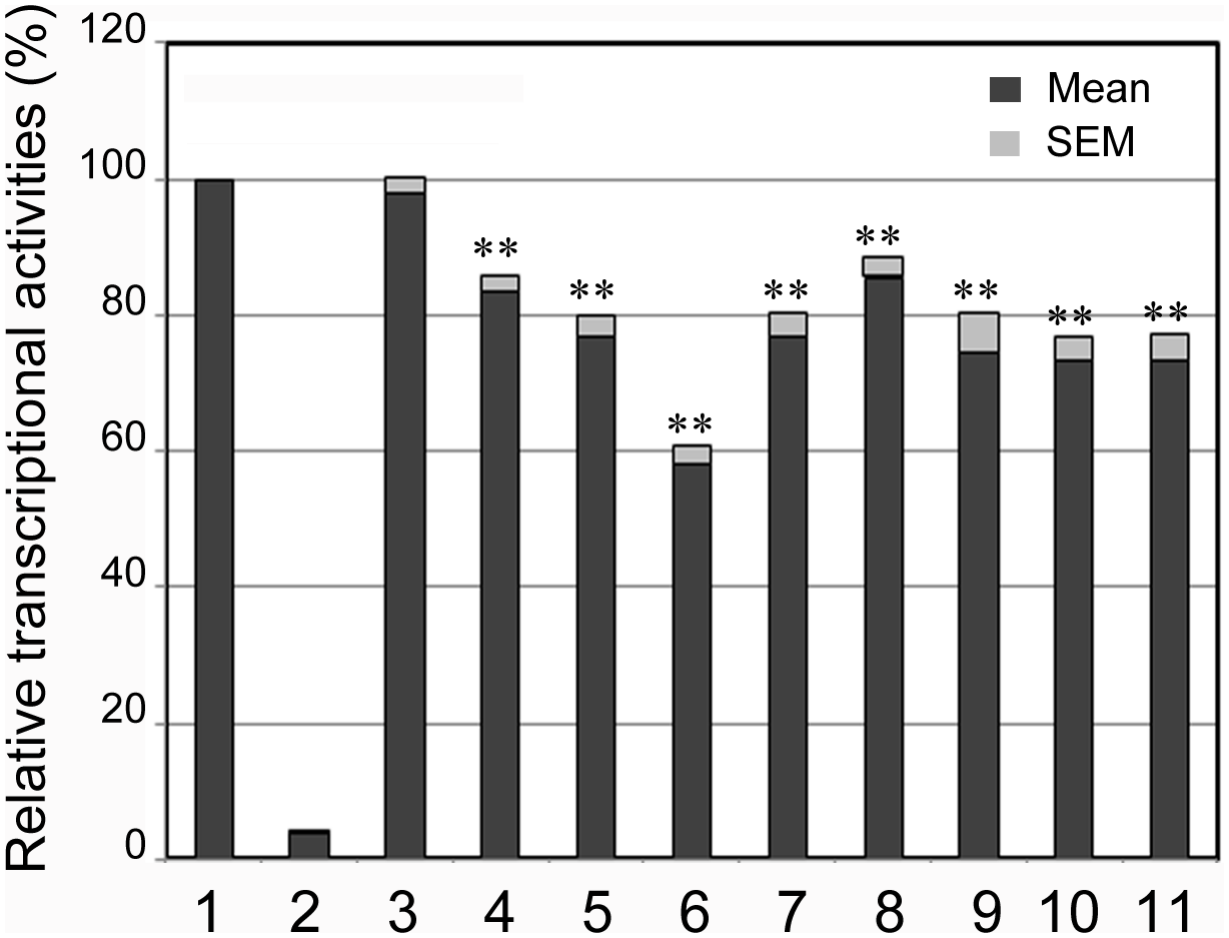
Relative transcriptional activities of wild type and variant SIRT3 gene promoters in HEK-293 cells. Expression constructs for wild type and variant SIRT3 gene promoters were transfected into HEK-293 cells and dual-luciferase activities were measured. Transcriptional activity of the wild type SIRT3 gene promoter was designed as 100%. Lanes 1, pGL3-WT; 2, pGL3 control; 3, pGL3-g.237312T>C (rs3817629); 4, pGL3- g.237307A>G; 5, pGL3- g.237270G>A + g.236370C>G + g.236380_81ins; 6, pGL3-g.237030C>T (rs12293349); 7, pGL3-g.237023_25del; 8, pGL3-g.237022C>G (rs369344513); 9, pGL3-g.236653C>A + g.236473C>T; 10, pGL3-g.236628G>C; 11, pGL3-g.236557T>C. **, P<0.01.

We further examined effects of the DSVs on the transcriptional activities of the SIRT3 gene promoter in HEK-293 cells. As shown in Fig 5, all the DSVs only identified in MI patients or the DSVs found in MI patients with higher frequencies, g.237307A>G, g.237270G>A+g.236370C>G+g.236380_81ins, g.237030C>T (rs12293349), g.237023_25del, g.237022C>G (rs369344513), g.236653C>A+g.236473C>T, g.236628G>C and g.236557T>C, significantly repressed the transcriptional activities of the SIRT3 gene promoter (P<0.05). The DSV g.236557T>C, which did not change the transcriptional activity of the SIRT3 gene promoter in H9c2 cells, significantly repressed the transcriptional activity of the SIRT3 gene promoter in HEK-293 cells, suggesting that the DSV may alter the binding site for a tissue-specific transcription factor. In addition, the DSV g.237312T>C (rs3817629), which was found in MI patients and controls with similar frequencies, did not change the transcriptional activities of the SIRT3 gene promoter (P>0.05).

## Discussion

To date, few studies have associated the SIRT3 gene with human diseases. A SNP rs11246020 (NC_000011.10:g.233067C>T) in the human SIRT3 gene, a nonsynonymous point mutation (V208I) which reduces its enzymatic activity, may enhance the susceptibility to metabolic syndrome [35]. A SNP rs11555236 (NC_000011.10:g.233212C>A) in intron 5 of the SIRT3 gene, which increases expression of SIRT3 gene, has been associated with extended lifespan of humans [7]. In this study, we have identified a total of 23 DSVs within the promoter of the human SIRT3 gene. Six novel heterozygous DSVs and two SNPs were identified in MI patients, but in none of controls (Table 2 and Fig 2). Three SNPs were found in MI patients with significantly higher frequencies compared to controls (Table 2). These DSVs and SNPs, except the DSV g.236557T>C, significantly decreased the transcriptional activities of the SIRT3 gene promoter in both H9c2 cells and HEK-293 cells (Figs 4 and 5). Therefore, these SIRT3 gene promoter DSVs may reduce SIRT3 levels, contributing to the MI development as risk factors.

The human SIRT3 gene is localized to the chromosome 11p15.5, and encodes an NAD-dependent mitochondrial deacetylase of 399-amino acids containing an N-terminal mitochondrial targeting signal and a central catalytic domain [40–42]. SIRT3 gene is expressed in a variety of tissues with higher expression in adipose tissue, brain and heart in embryos and adults (NCBI Unigene EST Profile Viewer), indicating the tissue-specific regulation of the SIRT3 gene expression. The human SIRT3 gene promoter contains high GC contents and lacks the TATA box sequence and there are binding sites for activator protein 1 (AP1), GATA-binding factor, nuclear Factor κB (NF-κB) and transcription factor ZF5, as well as multiple specificity protein 1 (SP1) binding sites [43]. Nuclear respiratory factor 2, a transcription factor that regulates mitochondrial genes, binds to the promoter of SIRT3 gene and induces its expression [44]. In this study, the DSVs reduced the SIRT3 promoter transcriptional activity in HEK-293 cells and H9c2 cells to different extents, which may be due to the tissue-specificity of the SIRT3 gene expression and related transcription factors. Therefore, expression of the human SIRT3 gene may be manipulated for therapeutic purposes.

A series of downstream substrates of SIRT3, as well as histone, have been identified. SIRT3 deacetylates and activates several enzymes that are critical in maintaining cellular ROS levels and promote resistance to oxidative stress, including superoxide dismutase 2 (SOD2) and isocitrate dehydrogenase 2 (IDH2) [12,45–49]. In experimental animals, SIRT3 deacetylates FOXO3a, a transcription factor that upregulates SOD2 and catalase, and decreases ROS levels [20]. Increased ROS levels and oxidative stress have been demonstrated to contribute to the atherogenesis [50,51]. In human endothelial cells, SIRT3 deacetylates and stabilizes FOXO3 to protect mitochondria against oxidative stress, and activates mitochondrial aldehyde dehydrogenase 2, a key enzyme to remove reactive aldehydes [24,52–54]. In the mitochondria, SIRT3 deacetylates acetyl-CoA synthase 2, 3-hydroxy-3-methylglutaryl CoA synthase 2, mitochondrial fusion protein optic atrophy 1 and regulatory component of the mitochondrial permeability transition pore, cyclophilin D [21,35,54]. SIRT3 has been shown to regulate long-chain acyl-CoA dehydrogenase, a key mitochondrial fatty acid oxidation enzyme [55]. SIRT3 regulates fatty acid oxidation through heat shock protein 10 [56]. In human diploid fibroblasts, SIRT3 overexpression antagonizes high glucose-induced cellular senescence via the SIRT3-FOXO1 signaling pathway [57]. A recent study has shown that SIRT3 targets human very long-chain acyl-CoA dehydrogenase, a key fatty acid oxidation enzyme [58]. Therefore, decreased SIRT3 levels may contribute to MI development by: 1) affecting lipid metabolism, inflammation and other pathways, initiating the atherosclerosis; and 2) interfering with fatty acid oxidation, ROS generation and mitochondrial functions, leading to death of cardiomyocytes. Exact molecular mechanisms need further investigated and elucidated.

## Conclusions

In this study, we genetically and functionally analyzed the SIRT3 gene promoter. The DSVs and SNPs of the SIRT3 gene promoter identified in MI patients may alter transcriptional activity of SIRT3 gene promoter and change SIRT3 level, contributing to the MI development as a risk factor. The investigation into the molecular mechanisms by which SIRT3 gene promoter activity are affected are being conducted in our laboratory. Therefore, our findings may provide a genetic basis for further translational and clinical studies for MI patients.
